# Perirenal Fat Thickness Is Associated With Contrast-Induced Nephropathy in Type 2 Diabetic Patients Undergoing Coronary Catheterization

**DOI:** 10.1155/2024/4905669

**Published:** 2024-08-22

**Authors:** Xixiang Tang, Jiafu Wang, Yuman Wu, Zhuoshan Huang, Xiaolan Ouyang, Hongxing Wu, Qian Chen, Junlin Zhong, Long Peng, Yan Lu, Bingyuan Wu, Yesheng Ling, Suhua Li

**Affiliations:** ^1^ VIP Medical Service Center Third Affiliated Hospital of Sun Yat-sen University, Guangzhou, China; ^2^ Department of Cardiovascular Medicine Third Affiliated Hospital of Sun Yat-sen University, Guangzhou, China; ^3^ Department of Clinical Immunology Third Affiliated Hospital of Sun Yat-sen University, Guangzhou, China; ^4^ Department of Ultrasonography Third Affiliated Hospital of Sun Yat-sen University, Guangzhou, China; ^5^ Guangdong Provincial Key Laboratory of Allergy & Clinical Immunology Second Affiliated Hospital of Guangzhou Medical University, Guangzhou, China

**Keywords:** contrast, coronary catheterization, diabetes, nephropathy, perirenal fat

## Abstract

**Background:** Deposition of adipose tissue may have a promoting role in the development of diabetic complications. This study is aimed at investigating the relationship between adipose tissue thickness and risk of contrast-induced nephropathy (CIN) in patients with Type 2 diabetes mellitus (T2DM).

**Methods:** A total of 603 T2DM patients undergoing percutaneous coronary angiography or angioplasty with suspicious or confirmed stable coronary artery disease were enrolled in this study. The thicknesses of perirenal fat (PRF), subcutaneous fat (SCF), intraperitoneal fat (IPF), and epicardial fat (ECF) were measured by color Doppler ultrasound, respectively. The association of various adipose tissues with CIN was analyzed.

**Results:** Seventy-seven patients (12.8%) developed CIN in this cohort. Patients who developed CIN had significantly thicker PRF (13.7 ± 4.0 mm vs. 8.9 ± 3.6 mm, *p* < 0.001), slightly thicker IPF (*p* = 0.046), and similar thicknesses of SCF (*p* = 0.782) and ECF (*p* = 0.749) compared to those who did not develop CIN. Correlation analysis showed that only PRF was positively associated with postoperation maximal serum creatinine (sCr) (*r* = 0.18, *p* = 0.012), maximal absolute change in sCr (*r* = 0.33, *p* < 0.001), and maximal percentage of change in sCr (*r* = 0.36, *p* < 0.001). In receiver operating characteristic (ROC) analysis, the area under the curve (AUC) of PRF (0.809) for CIN was significantly higher than those of SCF (0.490), IPF (0.594), and ECF (0.512). Multivariate logistic regression analysis further confirmed that thickness of PRF, rather than other adipose tissues, was independently associated with the development of CIN after adjusted for confounding factors (odds ratio (OR) = 1.53, 95% CI: 1.38–1.71, *p* < 0.001).

**Conclusions:** PRF is independently associated with the development of CIN in T2DM patients undergoing coronary catheterization.

## 1. Introduction

The administration of contrast media presents a significant risk in inducing contrast-induced nephropathy (CIN), which continues to be a leading contributor to acute kidney injury acquired during hospitalization [1]. Additionally, CIN is associated with extended hospitalizations and an elevated risk of chronic kidney disease (CKD), cardiovascular events, and mortality [2–4].

Among the nonrenal risk factors for CIN, diabetes bears the brunt. Patients with diabetes who undergo coronary catheterization have a significantly higher incidence of CIN, ranging from 20% to 30%, compared to the general population [5–7]. Approximately 50% of diabetic patients have CKD, which makes them more susceptible to CIN due to their vulnerable renal function. Nonetheless, the additional factors contributed to the elevated risk of CIN in diabetic patients have not yet been fully appreciated.

Obesity, characterized by excess adipose tissue, was often accompanied by diabetes and has generated significant interest regarding its potential association with CIN. However, the precise relationship between obesity and CIN remains controversial [8, 9]. Adipose tissue is composed of various depots, including subcutaneous fat (SCF) and visceral adipose tissue [10]. Recently, emerging evidence has highlighted the potential pathophysiological implications of different ectopic fat depots, such as perirenal fat (PRF), intraperitoneal fat (IPF), and epicardial fat (ECF), in cardiovascular and renal diseases [11]. Nevertheless, the associations between specific adipose tissue depots and the occurrence of CIN remain unknown. The present study was aimed at exploring the association of distinct adipose tissue depots and the development of CIN in a cohort of diabetic patients undergoing coronary catheterization.

## 2. Material and Methods

### 2.1. Study Population

This study is a single-center prospective cohort investigation carried out in the cardiology department of the Third Affiliated Hospital of Sun Yat-sen University. Inpatients with Type 2 diabetes mellitus (T2DM) undergoing percutaneous coronary angiography or angioplasty were enrolled from December 2020 to November 2021. The diagnosis of T2DM was in accordance with the 1999 criteria of the World Health Organization (WHO) [12]. Patients were slated for coronary catheterization upon meeting one of the specified criteria: (1) suspicious stable coronary artery disease based on angina-like symptoms, elevated cardiovascular risk, and abnormal clinical examinations (such as echocardiogram, stress ECG test, myocardial perfusion scintigraphy, and computed tomography coronary angiography); (2) verifying the patency of the bypass grafts or the stents in patients who had suspicious clinical manifestations after coronary artery bypass surgery or percutaneous coronary intervention; and (3) preoperative evaluation for noncoronary surgery. Subjects were excluded: (1) acute coronary syndrome (ACS); (2) any underlying condition associated with low effective blood volume; (3) various other types of CKDs; (4) conditions such as perirenal infection, large renal cysts, and other factors that may affect accurate measurement of PRF; (5) exposure to nephrotoxic drugs within 30 days prior to enrollment or throughout the study period; (6) allergy to contrast agent; (7) receiving dialysis; (8) breastfeeding or pregnancy; and (9) incomplete data for analysis. A total of 603 T2DM individuals were finally included. The study's protocol received approval from the Medical Ethics Committee of the Third Affiliated Hospital of Sun Yat-sen University, and all subjects have written the informed consents.

### 2.2. Data Collection

Baseline characteristics were acquired from all participants, including age, sex, smoking status, heart rate, blood pressure, body mass index (BMI), and medical history. Blood samples were collected following an 8-h fasting period prior to catheterization, and various biochemical parameters were subsequently analyzed using the Hitachi 7180 chemical analyzer (Hitachi High-Tech Corp, Japan): fasting blood glucose (FBG), lipid profile, uric acid, serum creatinine (sCr), and blood urea nitrogen (BUN). The estimated glomerular filtration rate (eGFR) based on the CKD-EPI equation was calculated [13]. Glycosylated hemoglobin A1c (HbA1c) was determined by high-performance liquid chromatography (Bio-Rad Laboratories, Inc., Hercules, CA, United States). Color Doppler echocardiography was performed as previously described [14] to obtain left ventricular ejection fraction (LVEF). Medications were also recorded.

### 2.3. Measurement of Adipose Tissues

The thicknesses of PRF, SCF, IPF, and ECF were determined by color Doppler ultrasound [15]. The thicknesses of PRF were measured in both kidneys of diabetic patients in the supine position, and the average value was taken. The probe was placed vertically on the skin on the outer surface of the patient's right abdomen, and an ultrasound longitudinal scan of the right kidney was performed, ensuring that the lateral edge of the kidney was aligned parallel to the skin surface. While capturing images, the minimal pressure on the probe was applied to ensure that high image resolution was achieved without compressing the adipose tissue. When measuring SCF, the maximum depth from the skin to the rectus abdominis was measured using a longitudinal scan at the end of exhalation at 1 cm above the umbilicus. IPF measurement was performed on the midline and left and right longitudinal lines of the longitudinal plane of the patient's abdomen (10 cm to the left and right of the midline, respectively). The average distance between the anterior border of the lumbar spine and the peritoneal border of the anterior abdominal wall was taken as the IPF thickness. When measuring ECF, two-dimensional transthoracic echocardiography was conducted on each subject, capturing images from standard parasternal and minor axis views. Maximum ECF thickness was assessed at the end-systole on the free wall of the right ventricle, perpendicular to the aortic annulus in the parasternal long-axis view, and perpendicular to the interventricular septum at both the midchordal and the tip of the papillary muscle levels in the parasternal short-axis view. The ECF thickness was determined by averaging the measurements from three cardiac cycles for each echocardiographic perspective. The measurements of all adipose tissue were independently conducted by two experienced echocardiographers in a blinded manner, and the mean of these values was determined. To evaluate the intra- and interobserve variability of the PRF thickness measurements, the intraclass correlation coefficient (ICC) was calculated. The thickness of PRF was measured twice and yielded an ICC of 0.958 (95% CI 0.908, 0.976; *p* < 0.001) for the reliability of the measurements. Two independent radiologists measured PRF thickness in 50 patients and evaluated interobserver variability. The ICC was 0.954 (95% CI 0.920, 0.974; *p* < 0.001), demonstrating high concordance between the two radiologists, with an ICC close to one indicating a strong correlation.

### 2.4. Coronary Catheterization and the Assessment of Incident CIN

All participants were administered prophylactic rehydration using 0.9% NaCl solution (1–2 mL/kg/h, intravenously) at 6 h prior to and 6 h following coronary catheterization. All antidiabetic medications (including metformin, sulfonylurea, and SGLT2i) would be continued during the procedure. Patients received either radial or femoral access for percutaneous coronary angiography or angioplasty, following standard clinical protocols. The type and dose of contrast medium were determined by two experienced interventional cardiologists depending on surgical needs. Dynamic changes of sCr values were estimated at baseline, as well as 24, 48, and 72 h following coronary catheterization. The primary outcome was the occurrence of CIN after coronary catheterization, which was identified as an increase in sCr level exceeding 25% or 0.5 mg/dL (≥ 44 *μ*mol/L) from baseline within 72 h following the administration of contrast agent, in the absence of other contributing factors [16].

### 2.5. Statistical Analysis

All statistical analyses were performed using SPSS software version 22.0 (SPSS, Chicago, IL, United States). Categorical variables were presented as percentages, while continuous variables were shown as means ± standard deviation. Differences in continuous variables between groups were assessed by Student's *t*-test, while differences in categorical variables were analyzed using the Pearson Chi-square test. Receiver operating characteristic (ROC) curves were drawn to detect the area under the curve (AUC) of different adipose tissues for predicting CIN. DeLong's test was employed to compare the AUC of various adipose tissues for predicting CIN using the MedCalc software. After checking the normality of distribution by Q-Q plot and Kolmogorov–Smirnov test, Pearson's or Spearman's correlations were used to explore the associations of the thicknesses of adipose tissues with pre- and postoperation kidney function. Univariate and multivariate logistic regression models were used to determine the association of different adipose tissues with the development of CIN. The odds ratio (OR) and 95% CIs were calculated based on per 1 mm change in PRF, SCF, IPF, and ECF. A two-tailed *p* value < 0.05 was considered to be statistically significant.

## 3. Results

### 3.1. Patient Characteristics

The baseline demographic and laboratory characteristics of the study participants are shown in Table 1. Among the 603 patients who underwent coronary catheterization, 77 (12.8%) developed CIN. Patients with CIN (refer as CIN [+]) exhibited lower eGFR levels (*p* = 0.021) and hemoglobin (*p* = 0.035), along with higher levels of sCr (*p* = 0.026), BUN (*p* = 0.002), uric acid (*p* = 0.019), and lipoprotein a (*p* = 0.020) compared to those without CIN (CIN [−]). There were no significant differences in other baseline characteristics between CIN (+) and CIN (−) patients.

**Figure 1:**
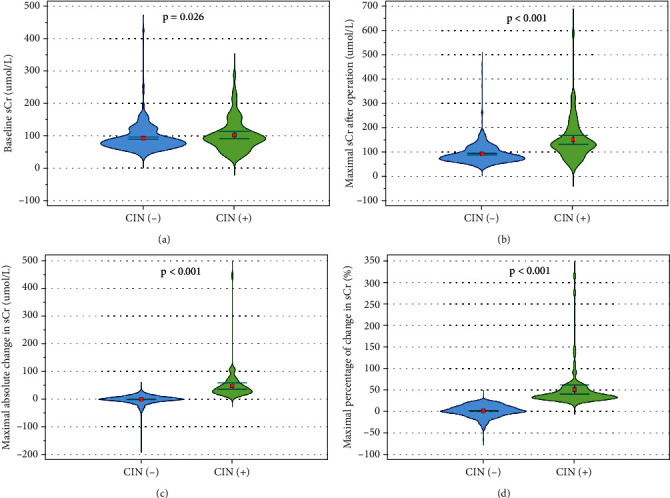
Pre- and postoperation serum creatinine in two groups. Compared to the CIN (−) group, the CIN (+) group had higher levels of (a) baseline sCr, (b) maximal sCr within 72 h after operation, (c) maximal absolute change in sCr, and (d) maximal percentage of change in sCr. CIN: contrast-induced nephropathy; sCr: serum creatinine.

Postoperation changes in sCr are shown in Figure 1. The maximal sCr level within 72 h after coronary catheterization in the CIN (+) group was significantly higher than that in the CIN (−) group (149.9 ± 81.6 vs. 92.2 ± 41.4 *μ*mol/L, respectively, *p* < 0.001). In addition, both the maximal absolute change in sCr (48.2 ± 51.9 vs. −0.22 ± 15.3 *μ*mol/L, respectively, *p* < 0.001) and the maximal percentage of change in sCr (49.8 ± 46.0% vs. 0.58 ± 13.3%, respectively, *p* < 0.001) after operation were also markedly greater in the CIN (+) group than those in the CIN (−) group.

### 3.2. Association of Adipose Tissues With Postoperation Kidney Injury

**Figure 2:**
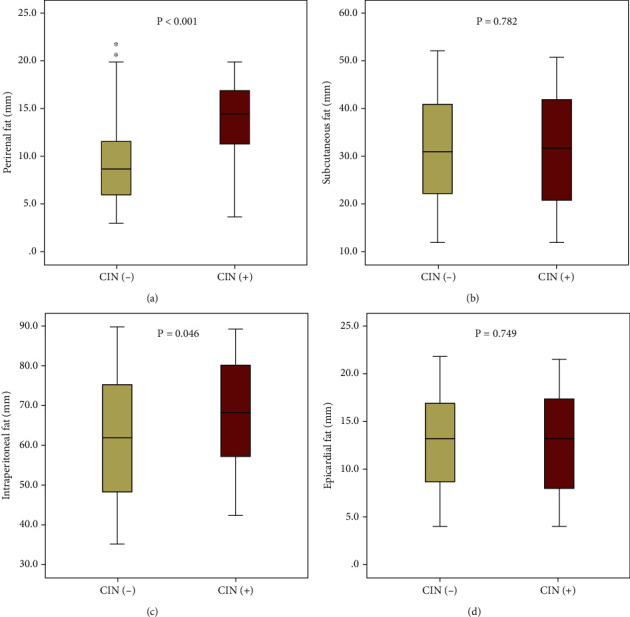
Thicknesses of different adipose tissues in two groups. Compared to the CIN (−) group, the CIN (+) group had significantly increased thickness of (a) perirenal fat, but only slightly higher thickness of (c) intraperitoneal fat. In addition, the thicknesses of (b) subcutaneous fat and (d) epicardial fat were similar between the two groups. CIN: contrast-induced nephropathy.

The thicknesses of adipose tissues in various regions were compared between the CIN (+) and CIN (−) groups (Figure 2). The thickness of PRF was remarkably greater in CIN (+) patients than in CIN (−) subjects (13.7 ± 4.0 mm vs. 8.9 ± 3.6 mm, respectively, *p* < 0.001). Additionally, the thickness of IPF was slightly higher in the CIN (+) group than that in the CIN (–) group (67.4 ± 13.8 vs. 62.4 ± 15.7, respectively, *p* = 0.046). Nevertheless, no significant differences were observed in the thicknesses of SCF (31.3 ± 11.8 vs. 31.7 ± 11.5, respectively, *p* = 0.782) and ECF (12.7 ± 5.4 vs. 12.9 ± 5.1, respectively, *p* = 0.749) between the two groups.

**Figure 3:**
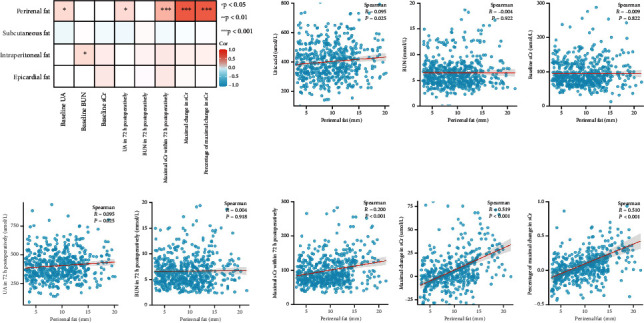
Correlations of different adipose tissues with pre- and postoperation renal function in diabetic patients. The thicknesses of all adipose tissues were not correlated with baseline sCr. However, the thickness of perirenal fat was positively associated with postoperation renal injury, while thicknesses of subcutaneous fat, intraperitoneal fat, and epicardial fat were not. sCr: serum creatinine; BUN: blood urine nitrogen; UA: uric acid.

Correlations of adipose tissues with pre- and postoperation kidney function were further analyzed in Figure 3. The thicknesses of all adipose tissues were not found to be correlated with baseline sCr in diabetic patients (all *p* > 0.05). However, the thickness of PRF was positively related with the maximal sCr level within 72 h after coronary catheterization (*r* = 0.182, *p* = 0.012), as well as the maximal absolute change in sCr (*r* = 0.327, *p* < 0.001) and the maximal percentage of change in sCr (*r* = 0.358, *p* < 0.001). Of note, no significant relationships were observed between the thicknesses of other adipose tissues (SCF, IPF, and ECF) and postoperation kidney injury in diabetic patients. In addition, only the thickness of PRF was positively associated with the level of uric acid in both baseline and 72 h postoperatively.

### 3.3. ROC Curve Analysis

**Figure 4:**
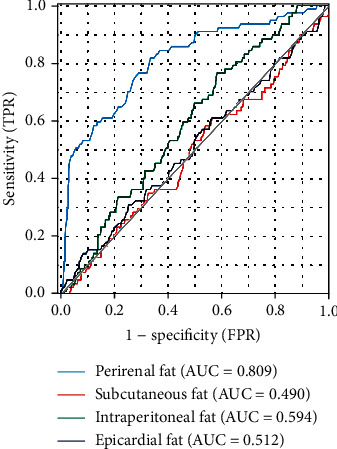
ROC curves of different adipose tissues for predicting CIN. The AUC of perirenal fat was dramatically higher than those of subcutaneous fat, intraperitoneal fat, and epicardial fat. ROC: receiver operating characteristic; AUC: area under the curve; CIN: contrast-induced nephropathy; TPR: true positive rate; FPR: false positive rate.

Figure 4 illustrates the ROC curves of different adipose tissues as predictors for CIN. Notably, the AUC of PRF (AUC = 0.809, 95% CI: 0.753–0.865) was significantly higher than the AUCs of SCF (AUC = 0.490, 95% CI: 0.419–0.561), IPF (AUC = 0.594, 95% CI: 0.531–0.656), and ECF (AUC = 0.512, 95% CI: 0.440–0.584) in predicting CIN. The AUC value of PRF was significantly higher than other types of adipose tissues (SCF, IPF, and ECF) (*Z* = 7.099, 5.105, 6.0576, respectively, all *p* < 0.01).

### 3.4. Logistic Regression Analysis

**Figure 5:**
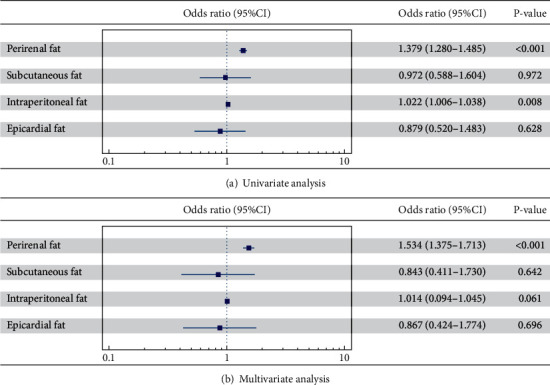
Logistic regression analysis assessing the association of different adipose tissues with the development of CIN. The multivariate analysis model was adjusted for age, gender, BMI, smoking status, concomitant diseases (hypertension, coronary artery disease, and congestive heart failure), baseline eGFR, FBG, HbA1c and lipid profiles, hemoglobin, uric acid, type of operation, type and dosage of contrast agent, pre- and postoperation hydratization, and medications (CCB, ACEI/ARB, *β*-blocker, statin, antiplatelet, diuretic, oral antidiabetic drug, and insulin). Odds ratios and 95% confidence intervals (CIs) were estimated for the association between the thicknesses of different adipose tissues and the development of CIN after operation. *p* < 0.05 was considered statistically significant. Results showed that among all adipose tissues, only the thickness of PRF could independently predict the development of CIN after adjusted for confounding factors. CIN: contrast-induced nephropathy.

In univariate logistic regression analysis (Figure 5(a)), per 1 mm increment in PRF was associated with a higher incidence of CIN (OR = 1.379, 95% CI: 1.280–1.485, *p* < 0.001). In addition, for per 1 mm increase in IPF, the risk of CIN slightly increased by 2.2% (OR = 1.022, 95% CI: 1.006–1.038, *p* = 0.008). However, no correlation was observed between the thicknesses of both SCF (*p* = 0.972) and ECF (*p* = 0.628) with the development of CIN.

Following adjustments for multiple confounding factors (including age, sex, BMI, smoking status, concomitant diseases, baseline eGFR, FBG, HbA1c and lipid profiles, hemoglobin, uric acid, type of operation, type and dosage of contrast agent, pre- and postoperation hydration, and medications), the multivariate logistic regression analysis (Figure 5(b)) further confirmed that only the PRF had a significant association with the onset of CIN after coronary catheterization (OR = 1.534, 95% CI: 1.375–1.713, *p* < 0.001), while the thicknesses of IPF (*p* = 0.061), SCF (*p* = 0.642), and ECF (*p* = 0.696) did not have any additional predictive value for the occurrence of CIN. These findings indicate that PRF might have a more pivotal role in the development of CIN compared to SCF, IPF, and ECF.

**Figure 6:**
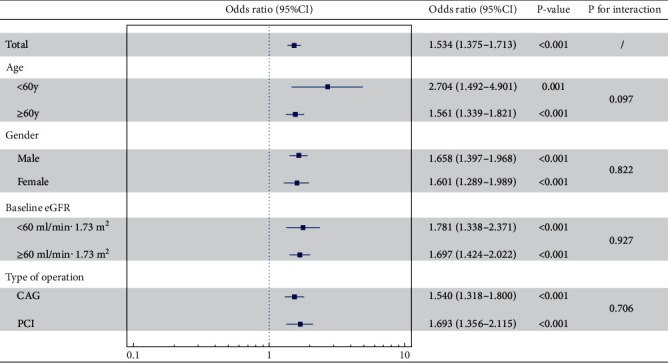
The relationship between PRF and CIN in subgroup analysis based on age, gender, baseline eGFR, and type of operation. The multivariate analysis models were used as mentioned in Figure 5. Odds ratios and 95% confidence intervals (CIs) were estimated for the association between the thickness of PRF and the development of CIN in different subgroup patients. *p* < 0.05 was considered statistically significant. Results showed that the thickness of PRF remained independently associated with CIN in all subgroups. PRF: perirenal fat thickness; CIN: contrast-induced nephropathy.

In subgroup analyses based on age, gender, baseline eGFR, and type of operation (Figure 6), we further found that the thickness of PRF remained independently linked to the onset of CIN in all subgroups (all *p* < 0.05). This suggests that the relationship between PRF thickness and the occurrence of CIN is not limited to specific patient subgroups.

## 4. Discussion

To the best of our knowledge, this is the first study to explore the relationship between various adipose tissues and the development of CIN. Consistent with our earlier abstract presented in the 34th Great Wall International Congress of Cardiology (GW-ICC)/Asian Heart Society (AHS) Congress 2023 [17], the current study provides more detailed research data and confirms that PRF, rather than fat deposits in other regions, was independently linked to the onset of CIN following coronary catheterization in T2DM patients. The thickness of PRF is a quick and simple predictor with considerable value that can help clinicians weigh the benefit, risk score, and stratification of exposure risk against diabetes before contrast administration, thereby facilitating the prevention of CIN.

In this study, the occurrence of CIN in T2DM participants who received coronary catheterization was 12.8%, which was higher than that (approximate 9%) reported in a recent meta-analysis of the general population [18]. However, previous retrospective studies have reported that the probability of CIN in diabetic patients could be as high as 20%–30% [5, 6, 19], which is considerably higher than the incidence observed in our study. This discrepancy may be attributed to the increasing standardization of diabetes management and variations in sample characteristics and hydration strategies across different research centers. Nevertheless, it is evident that both diabetes and contrast administration have a significant impact on renal physiology, including renal hemodynamics, tubular transport activity, oxygen depletion, increased medullary hypoxia, and ROS production. Diabetes can exacerbate these changes and disrupt protective mechanisms, leading to an increased susceptibility to CIN [20]. Therefore, it is crucial to identify high-risk patients and implement preventive measures, such as adequate hydration and the use of alternative imaging modalities, to minimize the risk of CIN in this vulnerable population.

PRF, as a part of visceral adipose tissue, is adjacent to the kidney in the retroperitoneal space between the renal fibrous membrane and the renal fascia. Recent studies have suggested that PRF works not only as a connective tissue that protects the kidney and renal vessels from external physical stimuli but also functions as a crucial endocrine organ that has links with metabolic disorders [21, 22], unfavorable profile of inflammatory cytokines [23, 24], renal calcified atherosclerosis [15], and the risk of CKD in diabetic subjects [25]. Recent studies have shown that PRF can sensitively reflect the glucose metabolism [26] and be strongly associated with urinary protein excretion and eGFR during the progression of diabetic kidney disease [27–29]. In this study, we found that an increase in PRF thickness is positively correlated with both the incidence of CIN and the postoperative severity of renal injury in diabetes. In addition, we found that only PRF independently predicted the occurrence of CIN, indicating that the local fat microenvironment of the kidney plays a key role in the target renal damage of diabetes. Compared to traditional predictors of CIN, PRF can be easily, quickly, and noninvasively measured by ultrasound to make its clinical application well-promoted, enabling risk prediction of CIN in patients with Type 2 diabetes. Combining PRF with other risk factors can lead to the development of a risk prediction model for CIN, providing clinical alerts and better monitoring and prevention measures for kidney function and hydration in these patients. Additionally, identifying the impact of drugs on PRF may aid in the development of drugs focused on preventing and treating CIN. Understanding the mechanism of how PRF affects kidney function can guide future clinical practice. Overall, these findings have important implications for preventing and treating CIN in clinical practice.

The mechanisms by which elevated PRF causes CIN in diabetic participants are not yet clear. There are several possible explanations. The development of CIN involves renal ischemia, inflammation, excessive ROS formation, decreased nitric oxide production, and damage to tubular epithelial and vascular endothelial cells [30]. PRF is connected to metabolic risk factors such as obesity, diabetes, hypertension, and dyslipidemia [21, 22]. This specialized type of visceral fat plays an active role in adipokine secretion and metabolism regulation [23, 24, 31–33]. Pathological conditions like diabetes can alter PRF's secretory profile, potentially increasing the risk of kidney disease. Previous animal studies have shown that the obese pigs with metabolic disorders had elevated levels of proinflammatory macrophages and TNF-a expression in PRF and more ROS in kidney compared to lean pigs [34–36]. The findings indicate that PRF could be involved in the progression of CIN among patients with diabetes.

Several limitations of this study should be considered. Firstly, this single-center study could limit the generalizability of our findings. Secondly, variations in the precautions were taken to prevent CIN before and after contrast administration, which may have influenced the incidence of CIN. Thirdly, the baseline renal function was worse in the patients with CIN, which potentially affects the development of CIN. Additionally, this is likely reflecting residual confounding rather than true causation between PRF and CIN in T2DM patients. Finally, the underlying molecular mechanisms linking PRF and CIN remain unclear, and further research is required to clarify this association to identify potential therapeutic targets.

## 5. Conclusions

In summary, PRF was found to be independently linked to the development of CIN in T2DM patients undergoing coronary catheterization. This study offers important perspectives on the prediction of CIN and serves as a useful addition to existing risk scoring models. Preoperative measurement of PRF can assist clinicians in assessing the risk of exposure relative to the benefits, conducting risk stratification, and preventing of CIN in diabetic patients.

## Figures and Tables

**Table 1:** Baseline characteristics.

**Characteristics**	**CIN (−) (** **N** = 526**)**	**CIN (+) (** **N** = 77**)**	**p** ** value**
Gender, *n* (%)			0.910
Male	345 (65.6)	50 (64.9)	
Female	181 (34.4)	27 (35.1)	
Age, year	64.1 ± 10.1	62.5 ± 11.3	0.202
BMI (kg/m^2^)	24.2 ± 3.5	23.8 ± 3.5	0.400
Smoker, *n* (%)	121 (23)	14 (18.2)	0.423
Duration of diabetes (year)	3 (2, 5)	3 (2, 5)	0.932
Hypertension, *n* (%)	358 (68.1)	53 (68.8)	0.996
Hyperlipidemia, *n* (%)	340 (64.6)	49 (63.6)	0.965
Coronary artery disease, *n* (%)	450 (85.6)	61 (79.2)	0.203
Congestive heart failure, *n* (%)	27 (5.1)	3 (3.9)	0.641
SBP (mmHg)	138.6 ± 22.5	141.6 ± 24.1	0.268
DBP (mmHg)	80.3 ± 12.3	80.3 ± 13.6	0.973
HR (bpm)	79.0 ± 14.0	79.1 ± 11.0	0.936
FBG (mmol/L)	7.65 ± 2.72	8.14 ± 3.79	0.162
HbA1c (%)	7.68 ± 1.87	7.76 ± 2.23	0.754
Hb (g/L)	131.0 ± 18.3	126.1 ± 23.1	0.035^∗^
UA (*μ*mol/L)	393.4 ± 116.1	434.9 ± 141.2	0.019^∗^
TC (mmol/L)	5.16 ± 1.35	5.35 ± 1.44	0.294
TG (mmol/L)	2.3 ± 1.63	2.23 ± 1.21	0.762
HDL-C (mmol/L)	1.92 ± 0.85	1.98 ± 0.84	0.602
LDL-C (mmol/L)	2.81 ± 1.05	3.02 ± 1.16	0.127
apoA1 (mmol/L)	1.55 ± 0.62	1.66 ± 0.72	0.191
apoB100 (mmol/L)	1.84 ± 0.81	1.77 ± 0.79	0.517
Lipoprotein a (mg/L)	259.8 ± 289.1	377.8 ± 390.6	0.020^∗^
BUN (mmol/L)	6.19 ± 2.61	7.94 ± 4.77	0.002^∗^
sCr (*μ*mol/L)	92.5 ± 41.1	101.7 ± 49.0	0.026^∗^
eGFR (mL/min/1.73 m^2^)	78.678 (56.688, 93.717)	71.402 (52.045, 94.088)	0.260
≥ 60 mL/min/1.73 m^2^	366 (69.6%)	47 (61%)	0.189
≥ 45, < 60 mL/min/1.73 m^2^	100 (19%)	16 (20.8%)	
< 45 mL/min/1.73 m^2^	60 (11.4%)	14 (18.2%)	
LVEF (%)	63.2 ± 12.6	63.3 ± 10.3	0.931
Type of operation, *n* (%)			
CAG	315 (59.9)	46 (59.7)	0.981
PCI	211 (40.1)	31 (40.3)	
Dosage of contrast agent (mL)	133.7 ± 61.6	139.3 ± 84.8	0.487
Type of contrast agent, *n* (%)			
Iopromide	85 (16.2)	13 (16.9)	0.997
Iodixanol (Westpike)	114 (21.7)	16 (20.8)	
Iohexol (Omnipaque)	47 (8.9)	7 (9.1)	
Iodixanol (others)	280 (53.2)	41 (53.2)	
Preoperation hydratization (mL)	684.9 ± 70.9	689.6 ± 74	0.588
Postoperation hydratization (mL)	746.3 ± 118.7	742.9 ± 109.6	0.811
Medication, *n* (%)			
CCB	212 (40.3)	36 (46.8)	0.342
ACEI/ARB	270 (51.3)	41 (53.2)	0.848
*β*-blocker	349 (66.3)	52 (67.5)	0.939
Statin	472 (89.7)	64 (83.1)	0.126
Antiplatelet	452 (85.9)	63 (81.8)	0.434
Diuretic	159 (30.2)	22 (28.6)	0.870
Oral antidiabetic drug	279 (53)	35 (45.5)	0.262
Insulin	170 (32.3)	23 (29.9)	0.765

*Note:* Data are mean ± SD, median (25th to 75th percentile), or *n* (%).

Abbreviations: ACEI/ARB: angiotensin-converting enzyme inhibitor/angiotensin receptor blocker; BMI: body mass index; BUN: blood urea nitrogen; CAG: coronary angiography; CCB: calcium channel blockers; CIN: contrast-induced nephropathy; DBP: diastolic blood pressure; eGFR: estimated glomerular filtration rate; FBG: fasting blood glucose; Hb: hemoglobin; HbA1c: hemoglobin A1c; HDL-C: high-density lipoprotein cholesterol; HR: heart rate; LDL-C: low-density lipoprotein cholesterol; LVEF: left ventricular ejection fraction; PCI: percutaneous coronary intervention; SBP: systolic blood pressure; sCr: serum creatinine; TC: total cholesterol; TG: triglycerides; UA: uric acid.

^∗^ *p* value < 0.05 was considered statistically significant.

## Data Availability

All data included in this study are available upon request by contact with the corresponding authors.

## Nomenclature

ACS: acute coronary syndrome
AUC: area under the curve
CIN: contrast-induced nephropathy
CKD: chronic kidney diseases
ECF: epicardial fat
eGFR: estimated glomerular filtration rate
ICC: intraclass correlation coefficient
IPF: intraperitoneal fat
LVEF: left ventricular ejection fraction
PRF: perirenal fat
ROC: receiver operating characteristic curve
SCF: subcutaneous fat
sCr: serum creatinine
T2DM: Type 2 diabetes mellitus
WHO: World Health Organization
